# Phylogeny, Genetic Diversity and Population Structure of *Fritillaria cirrhosa* and Its Relatives Based on Chloroplast Genome Data

**DOI:** 10.3390/genes15060730

**Published:** 2024-06-02

**Authors:** Jiao Huang, Xia Hu, Yong Zhou, Yan-Jie Peng, Zhong Liu

**Affiliations:** College of Life Science, Leshan Normal University, Leshan 614000, China

**Keywords:** *Fritillaria cirrhosa*, genetic diversity, population structure, phylogenetic relationship, chloroplast genome

## Abstract

*Fritillaria cirrhosa* and its relatives have been utilized in traditional Chinese medicine for many years and are under priority protection in China. Despite their medicinal and protective value, research on their phylogeny, genetic diversity, and divergence remains limited. Here, we investigate the chloroplast genome variation architecture of 46 samples of *F. cirrhosa* and its relatives collected from various regions, encompassing the majority of wild populations across diverse geographical areas. The results indicate abundant variations in 46 accessions including 1659 single-nucleotide polymorphisms and 440 indels. Six variable markers (*psbJ*, *ndhD*, *ycf1*, *ndhG*, *trnT-trnL*, and *rpl32-trnL*) were identified. Phylogenetic and network analysis, population structure analysis, and principal component analysis showed that the 46 accessions formed five clades with significant divergence, which were related to their geographical distribution. The regions spanning from the southern Hengduan Mountains to the Qinghai–Tibet Plateau exhibited the highest levels of genetic diversity. *F. cirrhosa* and its relatives may have suffered a genetic bottleneck and have a relatively low genetic diversity level. Moreover, geographical barriers and discrete patches may have accelerated population divergence. The study offers novel perspectives on the phylogeny, genetic diversity, and population structure of *F. cirrhosa* and its relatives, information that can inform conservation and utilization strategies in the future.

## 1. Introduction

*Fritillaria cirrhosa* is a perennial herbaceous flowering plant classified under the Liliaceae family and the *Fritillaria* genus. It is predominantly found across the regions of the Qinghai–Tibert Plateau (QTP) and Hengduan Mountains (HDM), as well as in the Himalaya and Qinba Mountains, typically in higher-altitude (3200–4600 m) regions. The bulbs of *F. cirrhosa* present important medicinal value and have been a cornerstone of traditional Chinese medicine for more than two millennia. They have antitussive, expectorant, anti-asthma, anticancer, antibacterial, and anti-inflammatory effects [1,2]. The morphological characteristics of *F. cirrhosa* show high variation due to its wide distribution range, forming a complex group that is currently divided into four species: *F. cirrhosa*, *F. sichuanica*, *F. yuzhongensis*, and *F. taipaiensis* [3]. Species that overlap or are distributed adjacent to the *F. cirrhosa* complex include *F. unibracteata*, *F. przewalskii*, *F. delavayi*, *F. crassicaulis*, *F. sinica*, *F. fusca*, and *F. dajinensis*, of which the first three have a wider distribution range and overlap more with the *F. cirrhosa* complex in terms of geographical distribution; the distribution of the last four is relatively narrow. The bulbs of *F. unibracteata*, *F. przewalskii*, *F. delavayi*, *F. taipaiensis*, and *F. unibracteata* var *wabuensis* are also classified as *F. cirrhosae bulbus* as per the Chinese Pharmacopoeia (2000 edition). Phylogenetic studies on *Fritillaria* have shown that the species of *Fritillaria* distributed near the QTP and HDM form a monophyletic clade with high support [4,5], but the evolutionary relationships of *F. cirrhosa* and its relatives remain unclear.

Owing to their high medicinal value, *F. cirrhosa* and its relatives have been excessively harvested, causing a significant decline in the populations of some species [6]. Furthermore, these species have lower fecundity due to their strict requirements for their growing environment [7]. All species of Chinese *Fritillaria* are included in the “Wild plants of national priority protection in China (Category-II), and *F. cirrhosa* is classified as “Vulnerable” on the IUCN Red List of threatened species. Consequently, genetic diversity forms the foundation for both germplasm conservation and the sustainable use of medicinal and threatened plants [8]. The genetic diversity observed within species and populations is a product of long-term evolutionary processes, and it is crucial for adaptation [9]. Understanding genetic diversity and population divergence will help in devising appropriate management policies and protective units [10]. In the past, various markers have been utilized in assessing the genetic diversity of *F. cirrhosa* and its relatives, such as amplified fragment length polymorphisms (AFLPs) [9,11] and inter-simple sequence repeats (ISSR) [12]. The corresponding results revealed the presence of numerous molecular differences among *F. cirrhosa* and its relatives, with widely distributed species showing greater genetic diversity compared to more localized species. However, these markers frequently offer limited insights into genetic variation within a population. The chloroplast genome presents a valuable source of information for genetic diversity studies.

The inheritance of the chloroplast genome is uniparental and shows a low recombination rate, while its rate of nucleotide substitution is moderate when compared to these rates for the nuclear or mitochondrial genomes [13,14]. The utilization of chloroplast genome sequences as DNA markers for understanding phylogenetic relations, across varying levels of divergence, has been widespread due to advancements in sequencing technology and genome assembly methods [14,15,16,17]. Whole chloroplast genomes contain numerous single-nucleotide polymorphisms (SNPs), insertion/deletion polymorphisms (indels), and single-sequence repeats (SSRs) at both the inter- and intra-species levels. These variations have been instrumental in characterizing genetic diversity and divergence in medicinal or endangered species [8,10,18], discerning population structure [17,19] and evaluating gene flow [20,21]. The phylogenomics of *Fritillaria* in China have been derived based on complete chloroplast genomes [5,22], indicating that DNA super barcodes can significantly improve species discriminatory resolution. Nevertheless, the level of variation within and between *F. cirrhosa* and its relatives remains unclear. Conducting assessments on multiple samples of species and genotypes may provide valuable insights into the population structures of *F. cirrhosa* and its relatives.

In this study, the chloroplast genomes of 31 samples of *F. cirrhosa* and its relatives were newly sequenced and assembled, along with 15 accessions that we sequenced previously and downloaded from GenBank, covering mostly wild distributions in China. Our objectives were as follows: (1) elucidate the variation in the chloroplast genome among *F. cirrhosa* and its relatives in China; (2) determine whether the pattern of chloroplast genome differentiation is consistent with species delimitation; and (3) assess the genetic diversity and population structure of *F. cirrhosa* and its relatives. The findings from this research will be beneficial for the conservation and utilization of *F. cirrhosa* and its relatives.

## 2. Materials and Methods

### 2.1. Taxon Sampling

**Figure 1:**
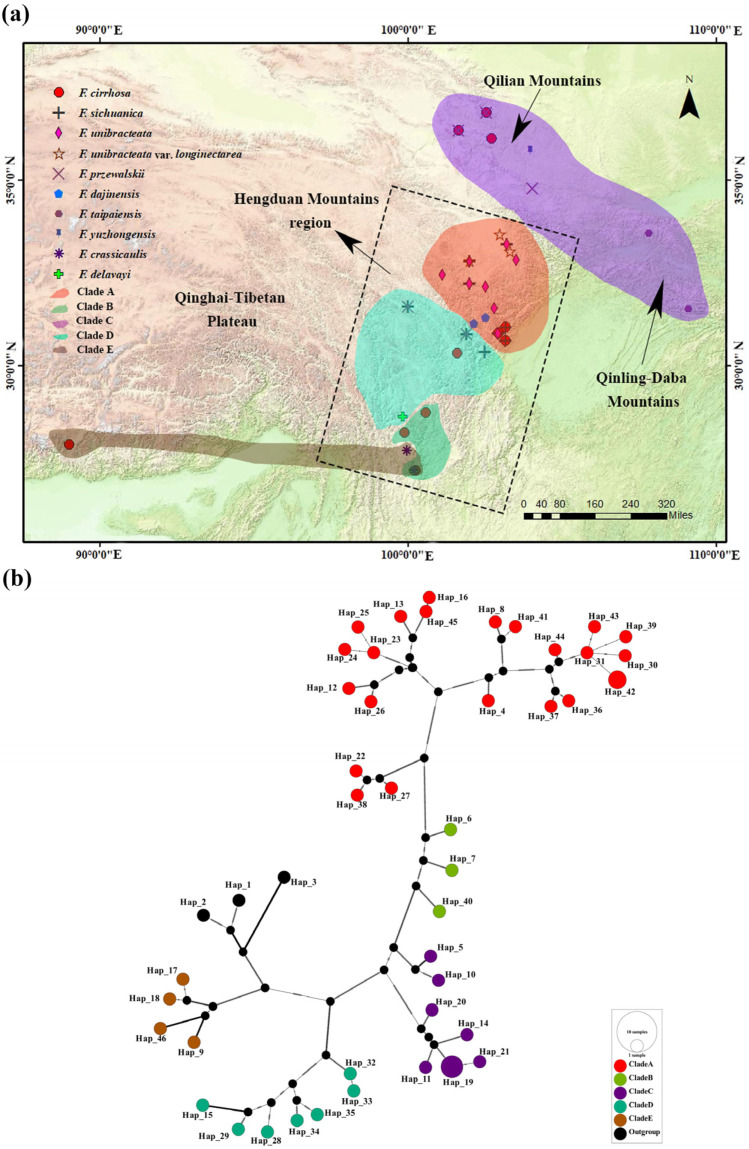
Distribution, sampling sites, and TCS network of *F. cirrhosa* and its relatives. (**a**) The various symbols represent different species and their distribution sites. The colors of the translucent blocks represent the clades to which the species belong, and the use of two colors for one distribution site indicates that the distribution is composed of two clades. Detailed sampling information is presented in Table S2. The topographic map is from https://www.tianditu.gov.cn/. URL (accessed on 5 February 2024) (**b**) TCS network of the chloroplast genome haplotypes for *F. cirrhosa* and its relatives and outgroups. The areas of the circles are proportional to the number of haplotypes. Missing haplotypes are indicated by black dots. Detailed information on the haplotypes is given in Table S4. Different colors of the circles (except for black, which represents outgroups) represent different phylogeographic clades and correspond to the color scheme employed throughout the figure.

The chloroplast genomes of 31 individuals from 18 populations were sequenced, and 15 accessions from 15 different populations were downloaded from GenBank (see Table S1 for detailed information on the individuals and populations and see Figure 1a for the population location); the samples of widespread species covered most of their geographic ranges, and the samples of narrow range species were collected to the type specimen sites. Fresh leaves from wild accessions were sampled in the field and subsequently dried using silica gel. The voucher specimens were identified by Jiao Huang and deposited at the herbarium of Leshan Normal University. All sequences were submitted to the Genbank database under accession numbers PP663650–PP663680.

### 2.2. Chloroplast Genome Sequencing, Assembly, and Gene Annotation

Total genomic DNA was isolated utilizing the magnetic bead technique, and the integrity of DNA was evaluated using agarose gel electrophoresis (1% *w*/*w*). The total DNA was sonicated to generate 350 bp fragments. Libraries for Illumina paired-end (PE) sequencing were prepared using the Illumina Novaseq X-plus platform, and the sequencing was performed using an Illumina genome analyzer (Hiseq PE150) to generate the original sequences. The removal of adaptor-contaminated reads, reads with quality values below Q20 for more than 50% of the bases, and reads with N proportions exceeding 5% resulted in obtaining clean reads.

SPAdes 3.14.1 program was utilized to conduct assembly of the clean data. Comparisons using Blastn and Exonerate were carried out utilizing the published chloroplast genome data of the genus *Fritillaria* and protein-coding gene (PCG) sequences, with set criteria of an e-value less than or equal to le-10 and a protein similarity threshold of 70%. Each gene-matched scaffold was carefully selected, and assembly coverage was arranged to eliminate fragments that clearly did not belong to the target genome. The tools PRICE 1.2 and MITObim (version 1.9.1) were employed to extensively combine and assemble the fragmented target sequence collected, with this process being repeated 50 times. Regarding the assembly results from the iterations, Bowtie2 (version 2.5.2) was deployed to inspect the original sequencing reads, selecting matching paired reads, and then SPAdes 3.15.5 was utilized for reassembly. The pathway was scrutinized, and a distinct circular map was chosen. If necessary, the iterative assembly and comparison procedures were reiterated until the circular map was successfully assembled. PGA software (2019) was employed to annotate the complete chloroplast genome, and the results were manually adjusted [23].

### 2.3. Variation Identification and Statistics

The chloroplast genome sequences of 31 accessions *F. cirrhosa* and its relatives were compared with 15 accessions downloaded from GenBank. Alignment was performed using MAFFT 7.49 [24], followed by manual adjustment using Se-al 2.0 [25]. Single-nucleotide polymorphisms and indels were analyzed to assess intra- and interspecific variation among 46 chloroplast genome accessions. SNPs and indels were calculated using DnaSP 6.12 [26] and MEGA 7.0 [27]. The position, number, and direction of SNPs and indels were determined using the QH01-01 genotype chloroplast genome as the standard reference. Nucleotide diversity (Pi) was calculated through sliding window analysis in DnaSP 6.12, utilizing a window length of 600 bp and step length of 200 bp.

### 2.4. Phylogenetic and Network Analysis 

The phylogenetic analyses in this study utilized three data sets, incorporating forty-six accessions from *F. cirrhosa* and its relatives, along with two accessions, i.e., *F. anhuiensis* (MK258148) and *F. monantha* (MK258143), and one accession, namely, *F. davidii* (MK258145), as outgroups. The three data sets were (1) the whole chloroplast genome (WCG) data set, which comprises the complete chloroplast genome; (2) the protein-coding gene (PCG) data set, which includes concatenated exons of protein-coding genes; and (3) the single-copy gene (SCG) data set. MAFFT v7.49 [24] was employed to ensure alignment with default parameters, and sequence pruning was accomplished using Gblocks. Phylogenetic consensus trees were constructed using both Bayesian inference (BI) and maximum likelihood (ML) methods. BI analyses were carried out with Mrbayes v3.2.6 [28], utilizing the Bayesian information criterion to select the best-fitting models. The BI analysis involved two hot and two cold chains run from random trees for ten million generations, sampling every 1000 generations and discarding the first 25% as burn-in. The final consensus tree was derived using the remaining trees with estimated posterior probabilities (PPs). ML analyses were implemented using IQ-TREE 2.0 software. The optimal model for ML was chosen via the IQ-TREE model finder based on the Bayesian criterion, with support values assessed through 1000 bootstrap replicates.

The chloroplast genome haplotypes (Table S2) were extracted from the WCG data set using DnaSP 6.12 [26], employing default parameters, and a TCS network was generated using PopART 1.7 [29].

### 2.5. Genetic Diversity and Population Differentiation

The population structures of 46 accessions were investigated using STRUCTURE v.2.3.4 [30]. The optimum number of clusters (K) was determined by applying the K-means clustering algorithm to a range of values from K = 2 to K = 10, each with 10 iterations. The length of the burning period was set to 10,000, followed by 100,000 MCMC Reps after burning. The DeltaK method of Structure Harvester was utilized to identify the most suitable clusters, the CLUMPP program was run for repeated sampling analysis, and distruct software was used to visualize the structure plots. Additionally, a principal component analysis (PCA) was conducted using PLINK 1.9 [31] to evaluate the genetic structure, and the resulting graphs were plotted using the ggbiplaot package within the R v4.1 statistical environment. 

To examine the genetic diversity among species and clades of *F. cirrhosa* and its relatives, several measures were calculated. The number of polymorphic sites (*S*), the number of haplotypes (*H*), haplotype diversity (Hd), and nucleotide diversity (Pi) in the WCG data set were determined using DnaSP 6.12 [26]. To assess the degree of sequence divergence within species and among clades, Arlequin 3.5 [32] was used to calculate Fu’ *Fs*, Tajima’s *D*, pairwise *F*_ST_, and AMOVA, and MEGA 7.0 [27] was employed to compute Nei’s *D*_A_ genetic distance. Because there was only one sample of *F. yuzhongensis*, it was not counted.

## 3. Results

### 3.1. Feature of the 31 Newly Sequenced Chloroplast Genomes 

A total of 31 complete chloroplast genomes of *F. cirrhosa* and its relatives were sequenced and annotated (Table S3). The gene arrangements found in all of these sequences were identical to those of *F. cirrhosa* [5]. The total length of the 31 chloroplast genome sequences was 150,949–152,103 bp, and the mean coverage was 124–697 (Table S3). Specifically, the large single-copy (LSC) region had a length of 81,257–81,868 bp, the single reverse repeat region ranged from 26,071 to 26,355 bp, and the small single-copy (SSC) region spanned from 17,527 to17,545 bp. The overall GC content was approximately 36.9–37.0%, with the IR region showing a higher GC content of 42.5–42.6% compared to the LSC (34.7–34.8%) and SSC regions (30.4–30.5%). In total, 131 genes were identified, consisting of 85 protein-coding genes, eight ribosomal RNA (rRNA) genes, and 38 transfer RNA (tRNA) genes.

### 3.2. Chloroplast Genome Sequence Variation 

**Figure 2:**
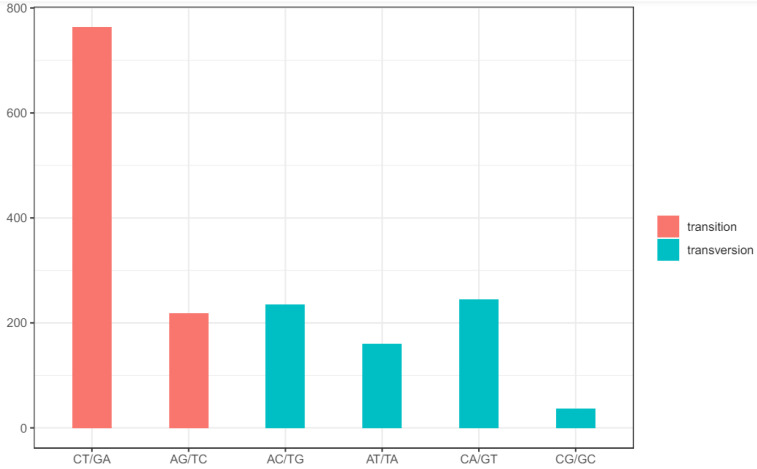
Patterns of SNPs among 46 chloroplast genome accessions. Nucleotide substitutions were divided into six types, as indicated by the six non-strand-specific base-substitution types (i.e., the number of G-to-A and C-to-T sites for each respective set of associated mutation types).

Integrating the chloroplast genomes of these specimens of *F. cirrhosa* and its relatives with those available in GenBank resulted in a total of 46 accessions (Table S1). The alignment sequences were 153,583 bp in length and contained 1659 SNP mutation sites, including 692 single-variable sites and 967 parsimony informative sites (Table 1), while the majority of SNPs were located in the LSC region (1178, 71.0%). Nucleotide diversity varied between the three different parts, ranging from 0.00039 in the IR region to 0.00288 in the SSC region, with an overall nucleotide diversity of 0.00159. Across the entire chloroplast genome, the average SNP density was 13.69/kb, with densities of 15.08/kb in the LSC, 17.12/kb in the SSC, and 7.10/kb in the IR region (Table 1). Genetic diversity, based on SNPs, was lower in the IR region compared to that in the LSC and SSC regions. Among the total SNP mutations, 773 mutations in spacer regions, 712 in coding regions, and 229 in intron regions were detected (Table S4). In the 712 coding-region SNPs, 369 were nonsynonymous SNPs. Thirteen genes were affected by >12 variant positions, and the most variable gene was *ycf1*, with 119 SNP sites, 90 of which were nonsynonymous SNPs, and the remaining 29 were synonymous SNPs. Ranking after *ycf1*, *ndhG* had 77 SNPs, and *ndhF* had 40 SNPs recorded (Table 2). Moreover, the gene *ndhG* exhibited the highest density of SNPs among the coding genes, with a rate of 144.19 SNPs per kb. The quantity and density of SNPs suggest a significant divergence in the coding genes *ycf1* and *ndhG* among the selected 46 identified accessions. The analysis of SNP patterns revealed a total of 982 transitions (Ts) and 677 transversions (Tv), resulting in an overall ratio of Ts:Tv of 1.451. The most frequent SNP mutation types were C to T and G to A, while C to G and G to C mutations were less frequent (Figure 2).

**Figure 3:**
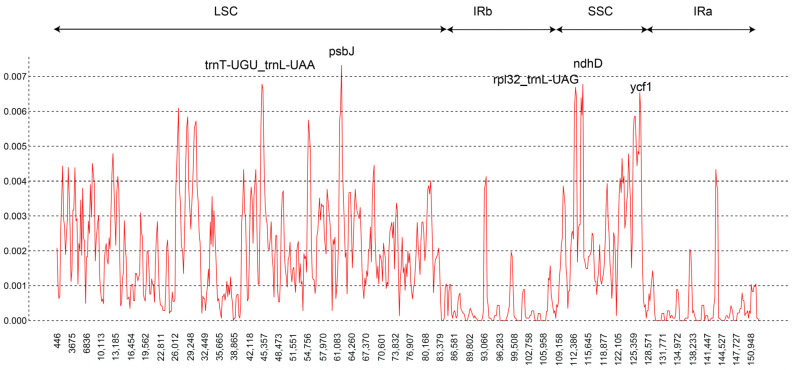
Sliding-window analysis of the whole chloroplast genomes of 46 accessions.

In the chloroplast genomes of 46 *F. cirrhosa* and its relatives, a total of 440 indels were discovered, most of which were located in noncoding regions (363 in LSC, 20 in IR regions, and 44 in SSC) (Table 1 and Table S4). The spacer *matK-rps16* showed the most indels, presenting 17, while *atpH-atpI* followed closely behind with 16 indels. In addition, *psbM-trnD*, *trnT-psbD*, *trnT-trnL*, *accD-psaI*, and the intron *rpl16* had >10 indels. Utilizing the sliding window method, we analyzed diversity hotspot regions within the whole chloroplast genomes of 46 accessions. A window size of 600 bp was employed for this analysis (Figure 3). The observed Pi values ranged from 0 to 0.0073. The lowest nucleotide diversity was found in the IR region. Five distinct peaks were identified, each associated with Pi values exceeding 0.0065. The five markers contained three coding regions (*psbJ*, *ndhD*, *ycf1*) and two intergenic regions (*trnT-trnL*, *rpl32-trnL*). *PsbJ* and *trnT-trnL* were situated in the LSC region, while the other three markers resided in the SSC region. The coding region *psbJ* harbored the highest Pi values (Pi = 0.0073).

### 3.3. Phylogenetic Relationships Based on the Chloroplast Genome

**Figure 4:**
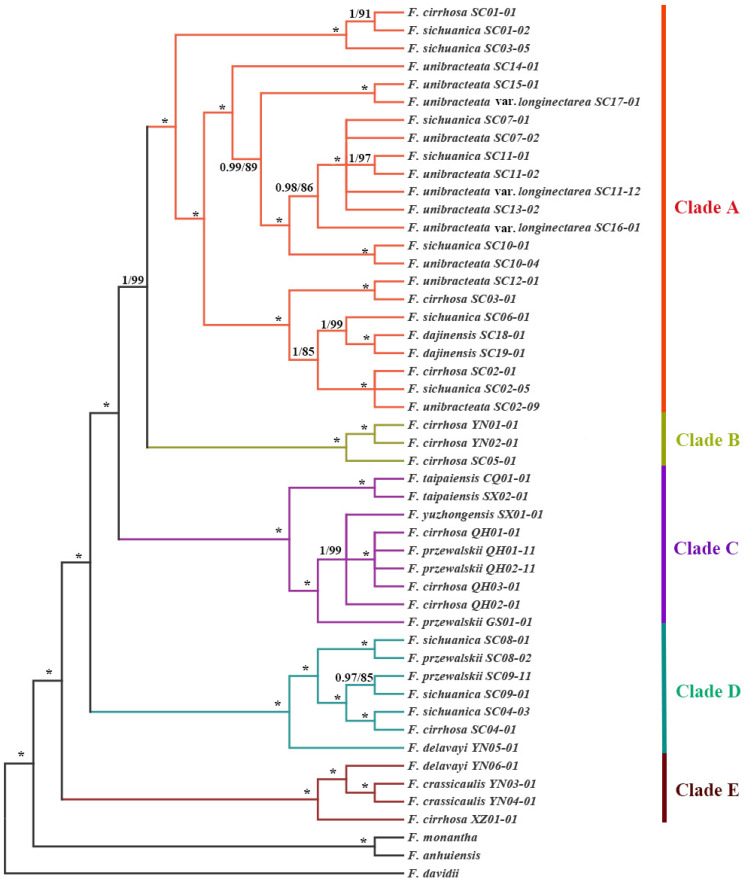
Phylogenetic relationship of 49 accessions inferred from BI and ML analyses based on the whole plastid genome data set. Support value markers above the branches correspond to PP (posterior probability)/BS (bootstrap support), and “*” indicates 100% support values in both BI and ML trees. The 46 accessions clustered into five clades which are indicated by different colors.

The phylogenetic analysis verified the monophyly of *F. cirrhosa* and its relatives, and their phylogenetic positions aligned with findings reported by Huang et al. [4,5]. The ML and BI trees constructed from the whole chloroplast genome (WCG) data set showed significant divergence, forming five phylogeographic clades with extremely high support values (Figure 4). These clades exhibited well-defined phylogeographic structures, with their distribution areas rarely overlapping, except for the overlaps occurring in the YN01, YN04, and YN06 populations (Figure 1a). These five clades also corresponded to the five groups defined by the haplotype network (Figure 1b). Furthermore, the topological structure of five clades is supported by phylogenetic trees derived from the protein-coding gene (PCG) and the single-copy gene (SCG) data sets (Figures S1 and S2). Evidently, the plastid genetic differentiation in *F. cirrhosa* and its relatives is correlated with their geographical distributions (Figure 1). Clade A mainly contains *F. unibracteata*, *F. unibracteata* var. *longinectarea*, *F. cirrhosa*, *F. sichuanica*, and *F. dajinensis* and is mainly distributed in the center and north of the Hengduan Mountains, including 22 haplotypes, among which SC11-01 and SC11-02 shared one haplotype (Table S2). Clade B was a sister to Clade A. Clade B contained *F. cirrhosa* and was primarily distributed in the south of the Hengduan Mountains, including three haplotypes. Clade C contained *F. taipaiensis*, *F. yuzhongensis*, *F. przewalskii*, and *F. cirrhosa* and was predominantly found in the Qilian Moutains and Qinling–Daba Mountains, including seven haplotypes, among which QH01-01, QH01-11, and QH02-11 shared one haplotype (Table S2). Clade D contained *F. przewalskii*, *F. cirrhosa*, *F. sichuanica*, and *F. delavayi*, which are mainly found in the central and southern regions of the Hengduan Mountains, including seven haplotypes. Clade E contained *F. crassicaulis*, *F. delavayi*, and *F. cirrhosa*, with a primary distribution ranging from the southern Hengduan Mountains to the Qinghai–Tibet Plateau, including four haplotypes.

### 3.4. Population Structure and PCA of F. cirrhosa and Its Relatives

**Figure 5:**
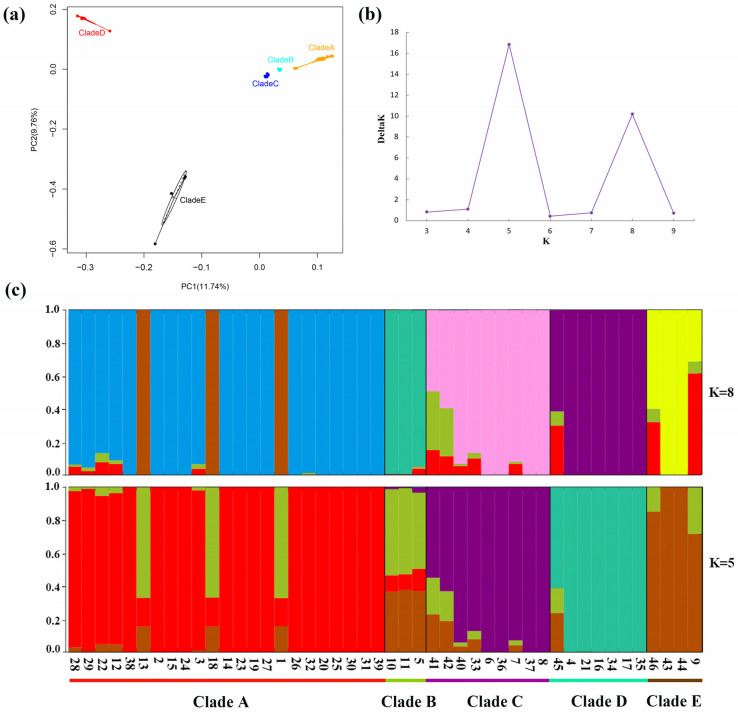
Population structure and PCA of *F. cirrhosa* and its relatives’ accessions. (**a**) PCA of all accessions. (**b**) The Delta K of STRUCTURE analysis. (**c**) Population structure clustering, with K = 5 and 8. Colors indicate different clusters. The *x*-axis shows the clades, numbers represent the individual numbers (see Table S1), and the *y*-axis indicates the probability of inferred ancestral lineages.

Using the whole-chloroplast genome sequences, a PCA revealed a clear differentiation of accessions, forming five distinct clades (Figure 5a). PC1 accounted for 11.74% of the variance, while PC2 explained 9.76%. PC1 and PC2 both suggested Clades A, B, and C had minor genetic differences. PC1 suggested Clade D had a genetic difference from Clades A, B, and C, despite the geographic proximity between Clade D and Clades A and B. PC2 suggested Clade E had genetic differences with Clades A, B, C, and D. Using the DeltaK method, we identified the most suitable “blood lineages” of *F. cirrhosa* and its relatives in China. The Structure Harvester analysis pinpointed the optimal value of K, that is, 5, with a secondary optimal value at K = 8 (Figure 5b). Based on the two suitable K-values, the resulting structure is presented in Figure 5c, showing the presence of five genetic groups within the 46 accessions. Specifically, when K = 5, individuals (1, 13, and 18—see Table S1) from Clade A were found to be largely assigned to the Clade B cluster, which was a genetic cross-cluster, and these individuals (1, 13, and 18—see Table S1) formed one subclade of Clade A with high bootstrap values in phylogenetic trees (Figure 4). The slight difference between the three individuals was also supported by the PCA and network analysis. The subclade including the three individuals was scattered from the rest of samples of Clade A in the PCA plot and network diagram. This suggests gene flow has occurred between some individuals of Clade A and Clade B, and there may be transitional groups between Clade A and Clade B.

### 3.5. Genetic Diversity in Different Clades and Species

The haplotype diversity (Hd) of the 46 WCG accessions was 0.996 (Table 3). Among the five genetic clades, Clade A contained 23 species including 22 haplotypes (Hd = 0.996), the highest number of any genetic clade, and the lowest Hd (0.917) was found in Clade C. The values of Pi were highest in Clade E (Pi = 0.00134) and lowest in Clade C (Pi = 0.00056). At the species level, *F. delavayi* displayed the highest Pi value (0.00248), followed by *F. cirrhosa* > *F. sichuanica* > *F. przewalskii* (Pi = 0.00168, 0.00146 and 0.00141, respectively), and the values of other species were small (Pi < 0.001) (Table 3). The haplotype diversity (Hd) of all species except *F. przewalskii* (Hd = 0.900) amounted to 1. Clade A, which included 486 SNPs and 226 indels, exhibited the highest diversity, while Clade B, with 172 SNPs and 60 indels, showed the lowest diversity levels (Table 4). The number of polymorphic sites (SNP) of *F. cirrhosa* was the highest, with the other corresponding to the following order: *F. sichuanica* > *F. przewalskii* > *F. delavayi* > *F. unibracteata*. The other species had a lower number of SNPs, all being less than 100 (Table 3). The values of Tajima’s *D* and Fu’*Fs* for the 46 WCG accessions were negative, with Tajima’s D being statistically significant (*p* < 0.05). This indicated that *F. cirrhosa* and its relatives in China have experienced positive selection pressure as a whole. Specifically, Clades A, C, D, and E along with *F. cirrhosa* and *F. unibracteata* exhibited signs of positive selection or population increases. On the other hand, *F. sichuanica* and *F. przewalskii* showed signs of balancing selection or population decreases. Clade B and other species were not subjected to any selective pressure.

The genetic differences (*F*_ST_) between the five clades ranged from 0.72577 to 0.51357 (Table 5) and were very significant (except for *F*_ST_ between Clade B and Clade E (*p* < 0.05)), indicating high genetic differentiation among the five clades. Analysis of molecular variance (AMOVA) produced similar conclusions, demonstrating that the majority of genetic diversity was present among clades (63.73%, *p* < 0.001) (Table 6).

## 4. Discussion

### 4.1. Inter- and Intraspecific Variation in the Chloroplast Genomes of F. cirrhosa and Its Relatives

The 31 newly sequenced chloroplast genomes of *F. cirrhosa* and its relatives will be crucial for identifying molecular markers and assessing genetic diversity. Through analyzing these sequences along with 15 others downloaded from GenBank, we identified genetic variations, including SNPs, indels, and divergence hotspot regions. A total of 1659 SNPs were found among the 46 accessions, with mutations primarily occurring from C to T or G to A based on base frequency. Conversely, C-to-G or G-to-C transversions were infrequent. The majority of SNPs were situated in the LSC regions, followed by SSC and IR regions, similar to the distribution of other mutaion types like indels. In general, non-coding regions exhibited higher SNP counts because they were asccoiated with less natural selection and fewer evolutionary constraints [10,33]. Notably, the genes *ycf1* and *ndhG* displayed a considerably higher number of SNPs compared to other genes. At the same time, five highly variable regions (*psbJ*, *ndhD*, *ycf1*, *rpl32-trnL*, and *trnT-trnL*) were identified within the 46 chloroplast genome accessions. The *ycf1* gene has been proposed to be a promising DNA barcode for angiosperms based on chloroplast genome data [34]. The phylogenetic analysis of the genus *Fritillaira* in China showed that the *ycf1* gene had great discrimination ability at the species level [22]. Furthermore, the intergenic spacer *trnT-trnL*, situated in the LSC region, has been extensively used for phylogenetic analysis and species identification [35]. Another intergenic spacer, *rpl32-trnL*, located in the SSC region, has been identified as a valuable marker by Shaw et al. [36] and Dong et al. [37], with both groups’ studies suggesting its efficacy in resolving closely related species in phylogenetic analyses due to its high variability compared to other chloroplast markers. The *ndhD* gene was also discovered in similar studies on *Fritillaria* [5,38]. Therefore, these six markers could serve as DNA barcodes for *F. cirrhosa* and its relatives due to their good performance.

Indels are common types of mutation events and potential informative phylogenetic characters in the chloroplast genome and can be used to reveal evolution and genetic variation [14,39]. In the 46 accessions of *F. cirrhosa* and its relatives, 440 indels were identified, which is less than the number of SNPs. The five intergenic spacers and one intron contained a higher number of indels, and *atpH-atpI*, *psbM-trnD, trnT-psbD*, *trnT-trnL*, and *rpl16* have been taken from the chloroplast genome for inter- and intraspecific phylogenetic studies [35,36]. Thus, these markers providing indel information can be used to improve the resolution of inter- and intraspecific phylogenetic studies.

### 4.2. Phylogenetic Relationships and Systematic Implications of F. cirrhosa and Its Relatives

The chloroplast genome data supported the monophyletic group of *F. dajinensis*, *F. taipaiensis,* and *F. crassicaulis*, with narrow ranges in this study. However, individuals from different populations of other species, mainly including *F. cirrhosa*, *F. sichuanica*, *F. przewalskii*, and *F. delavayi*, did not cluster together. The individuals of *F. unibracteata* and its variety *F. unibracteata* var. *longinectarea* were clustered into a large branch in which some individuals from other species were nested. This result was consistent with our previous studies based on plastid sequences (*matK*, *rpl16*, *rbcL*) and a combined plastid + nuclear ITS region (unpublished data). The four clades besides Clade B in the phylogenetic tree included taxa from multiple morphologically defined species, indicating a noticeable discordance between plastid distribution patterns and species distributions. Normally, after excluding inaccurate circumscriptions of species limits, such a scenario is often attributed to factors like incomplete lineage sorting (ILS) or hybridization/introgression [40,41]. Given that individuals in sympatric and adjacent areas in this study tended to share haplotypes, it is probable that hybridization/introgression occurs between species. A total of 46 haplotypes were obtained from the 46 chloroplast genomes analyzed in this study, among which only haplotype 19 and haplotype 42 are shared haplotypes (Figure 1; Table S2). Haplotype 42 is shared by *F. sichuanica* and *F. unibracteata* in the population from Aba County, Sichuan, China; Haplotype 19 is shared by *F. cirrhosa* and *F. przewalskii* in the populations from Huzhu County and Huangzhong District, Qinghai, China. This phenomenon suggests that plastid sharing among species and populations is primarily caused by plastid capture resulting from recent sympatric or adjacent hybridization. Due to the stochastic nature of lineage sorting in ancestral genotypes, it is unlikely for such a significant phenomenon of plasmid sharing among sympatric species to form [42,43]. The natural interspecific hybrids have been reviewed and reported in regard to *Fritillaria* [44], and species with the same distributions tend to be similar, such as *F. cirrhosa*, *F. przewalskii*, *F. sichuanica*, and *F. unibracteata* in the field. Therefore, this monophyletic clade (including *F. cirrhosa*, *F. przewalskii*, *F. sichuanica*, *F. unibracteata, F. unibracteata* var. *longinectarea, F. dajinensis, F. taipaiensis, F. crassicaulis*, *F. delavayi*, and *F. yuzhongensis*) should be called the *F. cirrhosa* complex, with hybridization/introgression possibly contributing to the intricate structure of the *F. cirrhosa* complex.

### 4.3. Phylogeographic Structure and Genetic Diversity of F. cirrhosa and Its Relatives

Our data clearly displayed significant genetic differentiation among five phylogeographic clades of *F. cirrhosa* and its relatives (Figure 4, Table 5), and the genetic differentiation among the five clades was higher than the genetic differentiation among populations within clades (Table 6). The development of these phylogeographic patterns could be attributed to geographic influences. Clade C predominantly inhabited the Qilian Moutains and Qinling–Daba Mountains, while Clades A, B, D, and E were primarily found in the area spanning from the Hengduan Mountains to the Qinghai–Tibet Plateau. The Qinling Mountains potentially act as a natural obstacle hindering gene flow between Clade C and the other four clades. The territory from the Hengduan Mountains to the Qinghai–Tibet Plateau is dotted with numerous high mountains and deep valleys, which serve as natural barriers whose presence leads to distinct vegetation patches. These barriers impede gene flow, fostering fragmentation and isolation, resulting in a relatively high level of genetic differentiation [45,46]. The cross-clustering of a few individuals in the five clades is likely an indication of infrequent long-distance seed or bulblet dispersal by *F. cirrhosa* and its relatives (Figure 5). The seeds of *Fritillaria* have flat and narrow wings, allowing them to travel via wind dispersal over some distance; on the other hand, bulblets are often dispersed underground by burrowing animals [4].

This research found that the genetic diversity of *F. cirrhosa* and its relatives (Pi = 0.00159) was relatively low [47], which is similar to the case for other threatened medicinal plants, such as *Angelica sinensis* [48], *Coptis chinensis* [49], and *Panax ginseng* [50]. Nevertheless, most of the samples of *F. cirrhosa* and its relatives had specific genotypes, and the level of haplotype diversity (Hd = 0.996) was relatively high [47]. This suggests *F. cirrhosa* and its relatives may have suffered a genetic bottleneck caused by human activity and geographical changes during the course of evolution and may be the result of a rapid population expansion after the bottleneck’s development. Our analyses of the whole chloroplast genome uncovered significant genetic variation across five clades and nine species (Table 3 and Table 4). Accessions in Clade E diverged earlier and had an extended evolutionary period compared to other clades, which might explain Clade E’s superior genetic diversity, yet the number of accessions of Clade E was relatively low. Similarly, *F. delavayi* had the fewest samples but presented the highest genetic diversity. This may be due to the fact that *F. delavayi* grows in special habitats at high altitudes and forms various genotypes [51].

### 4.4. Conservation Implications for F. cirrhosa and Its Relatives

Understanding genetic diversity and the patterns of genetic variation is essential for the conservation management of *F. cirrhosa* and its relatives [9,52]. This study has revealed that *F. cirrhosa* and its relatives have a low level of nucleotide diversity and that genetic bottlenecks were suffered during the course of their evolution, for especially *F. cirrhosa*, *F. unibracteata* (Table 3). Furthermore, *F. cirrhosa* and its relatives formed distinct phylogeographic structures, and it was observed that they have undergone higher genetic divergence. Considering the minimal amount of genetic diversity and the few genetic bottlenecks suffered, the germplasm resources of *F. cirrhosa* and its relatives must be strictly protected, and any further artificial over-exploitation should be rigorously banned. The regions spanning from the south of the HDM to the QTP may be conservation priority areas for *F. cirrhosa* and its relatives because of the maximal genetic diversity found there in this study. In addition, it is necessary to strengthen research on the species discrimination of *F. cirrhosa* and its relatives. Multiple sources of genomic data should be used to understand the complex interspecific relationships of *F. cirrhosa* and its relatives, and the hotspot regions proposed in this study could be used to clarify different genotypes in a more cost-effective way. More cultivated varieties need to be developed based on a distinct taxonomic basis in order to relieve the pressure on the demand for medicinal sources. 

## Figures and Tables

**Table 1:** Nucleotide diversity and variables of 46 chloroplast genome accessions.

Location	Polymorphic Sites	Singleton Variable Sites	Parsimony Informative Sites	Indels	Nucleotide Diversity	SNP Density/kb
IR	71	33	38	20	0.00039	7.10
LSC	1178	490	688	363	0.00209	15.08
SSC	324	122	202	44	0.00288	17.12
Total	1659	692	967	440	0.00159	13.69

**Table 2:** Highly variable chloroplast-protein-coding genes of 46 chloroplast genome accessions.

Gene	Protein	Length bp	Nn	Sn	SNP	Density of SNPs/kb
*ycf1*	hypothetical protein RF1	5523	90	29	119	21.55
*ndhG*	NADH-plastoquinone oxidoreductase subunit 6	534	66	11	77	144.19
*ndhF*	NADH-plastoquinone oxidoreductase subunit 5	2229	19	21	40	17.95
*rpoC2*	RNA polymerase β” subunit	4143	15	22	37	8.93
*matK*	maturase K	1539	16	12	28	18.19
*ndhD*	NADH-plastoquinone oxidoreductase subunit 4	1503	8	16	24	15.97
*ycf2*	hypothetical protein RF2	6654	17	2	19	2.86
*rps19*	ribosomal protein S19	279	15	4	19	68.1
*atpB*	ATP synthase CF1 β subunit	1497	3	14	17	11.36
*accD*	acetyl-CoA carboxylase carboxyltransferase β subunit	1464	9	8	17	11.61
*psbB*	photosystem II CP47 chlorophyll apoprotein	1527	3	14	17	11.13
*rpoB*	RNA polymerase β subunit	3207	8	7	15	4.68
*ndhA*	NADH-plastoquinone oxidoreductase subunit 1	1092	6	7	13	11.9

**Table 3:** Genetic diversity and neutrality test for five clades of 46 chloroplast genome accessions.

Group	*N*	*S*	*H*	Hd	Pi	Fu’ *Fs*	Tajima’s *D*
Whole samples	46	1659	43	0.996	0.00159	−0.22190	−1.39232 *
Clade A	23	486	22	0.996	0.00072	−0.54245	−0.62784
Clade B	3	172	3	1	0.00071	3.55221	0
Clade C	9	281	7	0.917	0.00056	5.17739	−0.67671
Clade D	7	272	7	1	0.00062	1.38102	−0.76924
Clade E	4	385	4	1	0.00134	3.49066	−0.35595
*F. cirrhosa*	11	980	11	1	0.00168	1.41309	−1.16719
*F. sichuanica*	10	580	10	1	0.00146	1.48401	0.41613
*F. unibracteata*	8	234	8	1	0.00051	0.84450	−0.71048
*F. unibracteata* var. *longinectarea*	3	72	3	1	0.00031	2.70083	0
*F. przewalskii*	5	410	4	0.900	0.00141	7.16923	0.97810
*F. dajinensis*	2	5	2	1	0.00003	1.60944	0
*F. taipaiensis*	2	72	2	1	0.00045	4.18965	0
*F. crassicaulis*	2	4	2	1	0.00003	1.38629	0
*F. delavayi*	2	395	2	1	0.00248	5.90536	0

Abbreviations: *H*, number of haplotypes; Hd, haplotype diversity; *N*, sample size; *S*, number of polymorphic sites; Pi, nucleotide diversity. * *p* < 0.05.

**Table 4:** Summary of the total variations (SNPs/indels) detected in the whole collection and in each clade.

Group	Accessions	All Variations			
		SNPs	Indels	Total	Density/kb
Clade A	23	486	226	712	4.70
Clade B	3	172	60	232	1.53
Clade C	9	281	109	390	2.57
Clade D	7	272	110	382	2.52
Clade E	4	385	115	500	3.30
Total	46	1659	440	2099	13.85

**Table 5:** Sequence divergence between five clades.

Comparison	*D*_A_	*F*_ST_ (*p*-Value)
Clade A vs. Clade B	0.0016235122	0.55553 (0.00000)
Clade A vs. Clade C	0.0017567195	0.62007 (0.00000)
Clade A vs. Clade D	0.0021522149	0.67751 (0.00000)
Clade A vs. Clade E	0.0022692043	0.61952 (0.00000)
Clade B vs. Clade C	0.0017149965	0.65073 (0.00000)
Clade B vs. Clade D	0.0020852724	0.68970 (0.00000)
Clade B vs. Clade E	0.0022138765	0.51357 (0.00592–0.03012)
Clade C vs. Clade D	0.0021363073	0.72577 (0.00000)
Clade C vs. Clade E	0.0022374106	0.63491 (0.00000)
Clade D vs. Clade E	0.0023211821	0.61595 (0.00000)

**Table 6:** The AMOVA results among and within clades.

	Source of Vatiation	Percentage of Variation
AMOVA	Among clades	63.73
	Within clades	36.27

## Data Availability

The data presented in this study are available on request from the corresponding author.

## Supplementary Materials

The following supporting information can be downloaded at https://www.mdpi.com/article/10.3390/genes15060730/s1. Table S1. Information on 46 samples for 33 populations of *Fritillaria cirrhosa* and its relatives. Table S2. Haplotypes based on the WCG data set. Table S3. Characteristics of the 31 newly sequenced chloroplast genomes. Table S4. Total number of SNP and Indel loci in 46 chloroplast genome accessions. Figure S1. Phylogenetic relationship of 49 accessions inferred from BI and ML analyses based on the protein-coding gene (PCG) data set. Support value markers above the branches correspond to PP (posterior probability)/BS (bootstrap support), and “*” indicates 100% support values in both BI and ML trees. The 46 accessions clustered into five clades which are indicated by different colors. Figure S2. Phylogenetic relationships of 49 accessions inferred from BI and ML analyses based on the single-copy gene (SCG) data set. Support value markers above the branches correspond to PP (posterior probability)/BS (bootstrap support), and “*” indicates 100% support values in both BI and ML trees. The 46 accessions clustered into five clades are indicated by different colors.
