# Supplementary material for: Phylogeny, Genetic Diversity and Population Structure of Fritillaria cirrhosa and Its Relatives Based on Chloroplast Genome Data
Source: Genes (Basel). 2024 Jun 2;15(6):730. doi: 10.3390/genes15060730 (PMC11202927; doi:10.3390/genes15060730)
Supplement: Supplementary file 1 [file genes-15-00730-s001.zip › Table S1.pdf]

Table S1. 46 samples information for 33 populations of *Fritillaria cirrhosa* and its relatives

Species	Population(s)/ No.	Individual(s)/ No.	Locality	Latitude(N)	Longitude(E)	Altitude(m)	Accession number
F. cirrhosa	SC01/1	SC01-01/1	Dayi County, Sichuan	103.1600	30.6900	3167	PP663671
F. cirrhosa	SC02/2	SC02-01/2	Xiaojin County, Sichuan	102.9197	30.8914	3851	PP663656
F. cirrhosa	SC03/3	SC03-01/3	Wenchuan County, Sichuan	103.1669	31.0541	3150	PP663654
F. cirrhosa	SC04/4	SC04-01/4	Kangding City, Sichuan	101.5847	30.3436	4200	PP663661
F. cirrhosa	SC05/5	SC05-01/5	Muli County, Sichuan	100.5586	28.6854	3600	PP663673
F. cirrhosa	QH01/6	QH01-01/6	Huzhu County, Qinghai	102.5467	36.7503	2791	PP663650
F. cirrhosa	QH02/7	QH02-01/7	Huangzhong District, Qinghai	101.6278	36.3025	3323	PP663652
F. cirrhosa	QH03/8	QH03-01/8	Minhe County, Qinghai	102.7139	36.0869	3787	PP663654
F. cirrhosa	XZ01/9	XZ01-01/9	Yadong County, Xizang	88.9926	27.8008	4173	PP663680
F. cirrhosa	YN01/10	YN01-01/10	Lijiang City, Yunnan	100.2355	27.0595	3142	MH593342
F. cirrhosa	YN02/11	YN02-01/11	Shangri-La City, Yunnan	99.8813	28.1350	4212	MH593344
F. sichuanica	SC06/12	SC06-01/12	Baoxing County, Sichuan	102.4848	30.3858	4050	MK258141
F. sichuanica	SC01/1	SC01-02/13	Dayi County, Sichuan	103.1600	30.6900	3167	PP663655
F. sichuanica	SC07/13	SC07-01/14	Ruoergai County, Sichuan	103.2021	33.3208	3779	PP663663
F. sichuanica	SC02/2	SC02-05/15	Xiaojin County, Sichuan	102.9197	30.8914	3851	PP663657
F. sichuanica	SC08/14	SC08-01/16	Ganzi County, Sichuan	99.9931	31.6267	4060	PP663665
F. sichuanica	SC09/15	SC09-01/17	Danba County, Sichuan	101.8964	30.8848	4300	PP663667
F. sichuanica	SC03/3	SC03-05/18	Wenchuan County, Sichuan	103.1669	31.0541	3150	PP663660
F. sichuanica	SC10/16	SC10-01/19	Barkam City, Sichuan	101.9900	32.2560	3200	PP663669
F. sichuanica	SC11/17	SC11-01/20	Aba County, Sichuan	101.9833	32.8500	3480	PP663675
F. sichuanica	SC04/4	SC04-03/21	Kangding City, Sichuan	101.5847	30.3436	4200	PP663662
F. unibracteata	SC12/18	SC12-01/22	Li County, Sichuan	102.7997	31.5904	3600	MK258140

F. unibracteata	SC07/13	SC07-02/23	Ruoergai County, Sichuan	103.2021	33.3208	3779	PP663664
F. unibracteata	SC02/2	SC02-09/24	Xiaojin County, Sichuan	102.9197	30.8914	3851	PP663658
F. unibracteata	SC11/17	SC11-02/25	Aba County, Sichuan	101.9833	32.8500	3480	PP663676
F. unibracteata	SC13/19	SC13-02/26	Zamtang County, Sichuan	101.1205	32.4958	4200	PP663672
F. unibracteata	SC10/16	SC10-04/27	Barkam City, Sichuan	101.9900	32.2560	3200	PP663670
F. unibracteata	SC14/20	SC14-01/28	Hongyuan County, Sichuan	102.5114	32.1755	3621	MH244909
F. unibracteata	SC15/21	SC15-01/29	Songpan County, Sichuan	103.5065	32.8903	3199	MH593351
F.unibracteata var. *longinectarea*	SC11/17	SC11-12/30	Aba County, Sichuan	101.9833	32.8500	3480	PP663677
F.unibracteata var. *longinectarea*	SC16/22	SC16-01/31	Ruoergai County, Sichuan	102.9618	33.5759	3500	PP663678
F.unibracteata var. *longinectarea*	SC17/23	SC17-01/32	Jiuzhaigou County, Sichuan	103.303797	33.108817	4300	PP663674
F. przewalskii	GS01/24	GS01-01/33	Zhang County, Gansu	104.0372	34.7919	3145	MK258142
F. przewalskii	SC08/14	SC08-02/34	Ganzi County, Sichuan	99.9931	31.6267	4060	PP663666
F. przewalskii	SC09/15	SC09-11/35	Danba County, Sichuan	101.8964	30.8848	4300	PP663668
F. przewalskii	QH01/6	QH01-11/36	Huzhu County, Qinghai	102.5467	36.7503	2791	PP663651
F. przewalskii	QH02/7	QH02-11/37	Huangzhong District, Qinghai	101.6278	36.3025	3323	PP663653
F. dajinensis	SC18/25	SC18-01/38	Jinchuan County, Sichuan	102.1285	31.1636	3900	MK258146
F. dajinensis	SC19/26	SC19-01/39	Xiaojin County, Sichuan	102.5084	31.3211	4200	PP663679
F. yuzhongensis	SX01/27	SX01-01/40	Yuzhong County, Shaanxi	103.9713	35.7834	3210	MK258139
F. taipaiensis	CQ01/28	CQ01-01/41	Wuxi County, Chongqing	109.1082	31.5643	2230	MH244910
F. taipaiensis	SX02/29	SX02-01/42	Foping County, Shaanxi	107.8079	33.6117	1470	MH593357
F. crassicaulis	YN03/30	YN03-01/43	Shangri-La City, Yunnan	99.9578	27.6255	4000	MK258147
F. crassicaulis	YN04/31	YN04-01/44	Lijiang City, Yunnan	100.1938	27.0513	3766	MN810998

F. delavayi	YN05/32	YN05-01/45	Shangri-La City, Yunnan	99.8335	28.5794	4550	MK258144
F. delavayi	YN06/33	YN06-01/46	Lijiang City, Yunnan	100.1968	27.0587	4071	MH593353
