# Supplementary material for: Phylogeny, Genetic Diversity and Population Structure of Fritillaria cirrhosa and Its Relatives Based on Chloroplast Genome Data
Source: Genes (Basel). 2024 Jun 2;15(6):730. doi: 10.3390/genes15060730 (PMC11202927; doi:10.3390/genes15060730)
Supplement: Supplementary file 1 [file genes-15-00730-s001.zip › Table S3.pdf]

Table S3. Characteristics of the 31 newly sequenced chloroplast genomes.

No.	Individuals	Species	Mean coverage	Sequence Length/bp	GC content (%)	LSC		IR		SSC	
						Sequence Length/bp	GC content (%)	Sequence Length/bp	GC content (%)	Sequence Length/bp	GC content (%)
1	SC01-01	*F. cirrhosa*	124	151774	37.0	81540	34.8	26350	42.5	17534	30.4
2	SC02-01	*F. cirrhosa*	648	151986	37.0	81762	34.8	26342	42.5	17540	30.4
3	SC03-01	*F. cirrhosa*	512	151979	36.9	81739	34.8	26350	42.5	17540	30.4
4	SC04-01	*F. cirrhosa*	345	152036	36.9	81798	34.8	26350	42.5	17538	30.4
5	SC05-01	*F. cirrhosa*	620	151795	37.0	81649	34.8	26301	42.5	17544	30.4
6	QH01-01	*F. cirrhosa*	697	151593	37.0	81364	34.8	26351	42.5	17527	30.4
7	QH02-01	*F. cirrhosa*	336	151667	37.0	81427	34.8	26355	42.5	17530	30.4
8	QH03-01	*F. cirrhosa*	290	151597	37.0	81368	34.8	26351	42.5	17527	30.4
9	XZ01-01	*F. cirrhosa*	303	151610	37.0	81385	34.8	26340	42.5	17545	30.4
10	SC01-02	*F. sichuanica*	272	151779	37.0	81540	34.8	26350	42.5	17539	30.4
11	SC07-01	*F. sichuanica*	646	151073	37.0	81394	34.8	26072	42.6	17535	30.4
12	SC02-05	*F. sichuanica*	308	151996	37.0	81770	34.8	26342	42.5	17542	30.4
13	SC08-01	*F. sichuanica*	479	152051	36.9	81811	34.8	26350	42.5	17540	30.4
14	SC09-01	*F. sichuanica*	483	152061	36.9	81824	34.8	26350	42.5	17537	30.5
15	SC03-05	*F. sichuanica*	408	151791	37.0	81552	34.8	26350	42.5	17539	30.4
16	SC10-01	*F. sichuanica*	420	151101	37.0	81420	34.8	26071	42.6	17539	30.4
17	SC11-01	*F. sichuanica*	381	151083	37.0	81390	34.8	26078	42.6	17537	30.4
18	SC04-03	*F. sichuanica*	194	152103	36.9	81868	34.7	26350	42.5	17535	30.5
19	SC07-02	*F. unibracteata*	304	151076	37.0	81384	34.8	26078	42.6	17536	30.4
20	SC02-09	*F. unibracteata*	361	151991	37.0	81766	34.8	26342	42.5	17541	30.4
21	SC11-02	*F. unibracteata*	219	151084	37.0	81391	34.8	26078	42.6	17537	30.4
22	SC13-02	*F. unibracteata*	245	151068	37.0	81377	34.8	26078	42.6	17535	30.4

23	SC10-04	*F. unibracteata*	332	151095	37.0	81411	34.8	26071	42.6	17542	30.4
24	SC11-12	*F. unibracteata* var. *longinectarea*	690	150949	37.0	81257	34.8	26078	42.6	17536	30.4
25	SC16-01	*F. unibracteata* var. *longinectarea*	179	151070	37.0	81393	34.8	26071	42.6	17535	30.4
26	SC17-01	*F. unibracteata* var. *longinectarea*	662	151014	37.0	81297	34.8	26090	42.6	17537	30.4
27	SC08-02	*F. przewalskii*	527	152038	36.9	81799	34.8	26350	42.5	17539	30.4
28	SC09-11	*F. przewalskii*	232	152059	36.9	81823	34.8	26350	42.5	17536	30.5
29	QH01-11	*F. przewalskii*	284	151593	37.0	81364	34.8	26351	42.5	17527	30.4
30	QH02-11	*F. przewalskii*	458	151595	37.0	81366	34.8	26351	42.5	17527	30.4
31	SC19-01	*F. dajinensis*	172	151788	37.0	81546	34.8	26350	42.5	17542	30.4
