# Supplementary figures and images for: Phylogeny, Genetic Diversity and Population Structure of Fritillaria cirrhosa and Its Relatives Based on Chloroplast Genome Data
Source: Genes (Basel). 2024 Jun 2;15(6):730. doi: 10.3390/genes15060730 (PMC11202927; doi:10.3390/genes15060730)

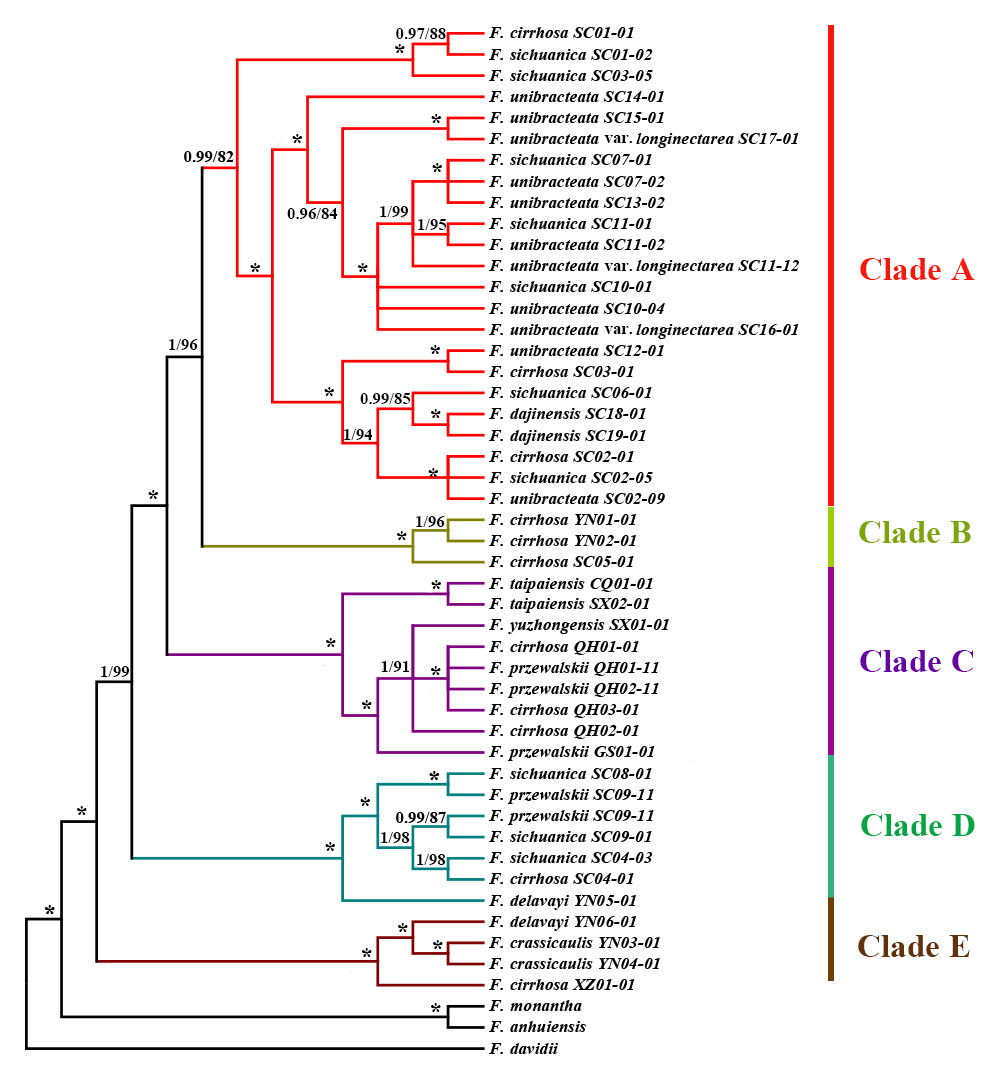

Supplement: Supplementary file 1 [file genes-15-00730-s001.zip › Figure S1.jpg]

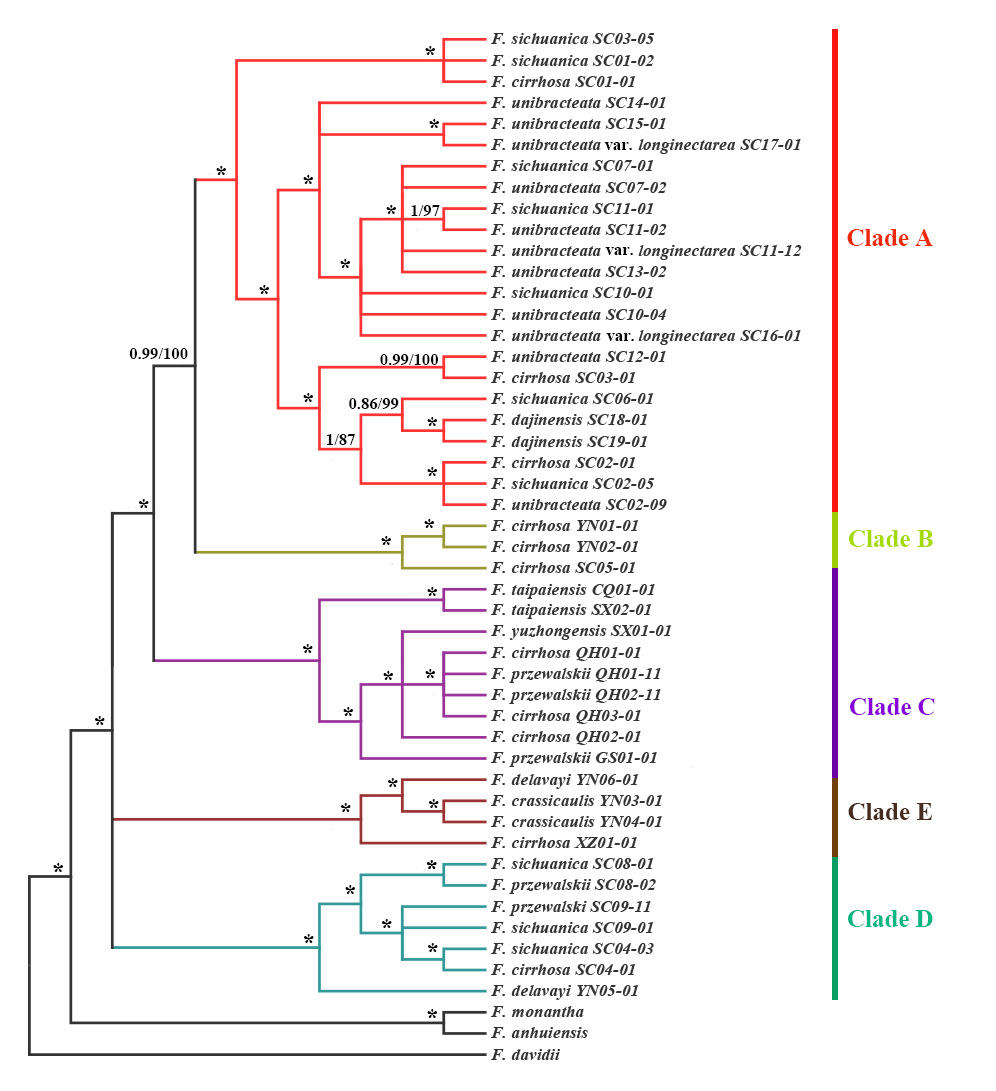

Supplement: Supplementary file 1 [file genes-15-00730-s001.zip › Figure S2.jpg]
